# Polymyxin B-induced rhabdomyolysis: A case report

**DOI:** 10.1097/MD.0000000000022924

**Published:** 2020-10-23

**Authors:** Ming Ni, Xiangdong Meng, Limin Wang, Yanan Zhao, Min Yu, Sheng Shi

**Affiliations:** a Department of Clinical Pharmacy, Henan Provincial People's Hospital; b Department of Clinical Pharmacy, Fuwai Central China Cardiovascular Hospital, Zhengzhou; c Department of Cardiovascular Surgery, Shanghai General Hospital, Shanghai Jiao Tong University, School of Medicine, Shanghai; d Department of Clinical Pharmacy, Henan Provincial Chest Hospital, Zhengzhou, China.

**Keywords:** drug adverse reaction, polymyxin B, rhabdomyolysis

## Abstract

**Rationale:**

Polymyxin B has been used to treat extensively drug-resistant gram-negative bacteria and shown a better antibacterial effect in the clinic at present. Meanwhile, polymyxin B is associated with several adverse effects. However, there is a lack of awareness that polymyxin B can cause rhabdomyolysis. In this study, we firstly report a case of polymyxin B-induced rhabdomyolysis during antiinfection therapy.

**Patient concerns:**

A 70-year-old woman suffering from rheumatic heart disease underwent aortic and mitral valve replacement at our institute. Subsequently, she developed bacteremia and pneumonia caused by extensively drug resistance-acinetobacter baumannii. Polymyxin B was administered for 5 days. During treatment, the patient complained of muscle pain and limb weakness, and her serum creatine phosphokinase and myoglobin levels rose.

**Diagnosis:**

The clinical symptoms and laboratory examination confirmed rhabdomyolysis, and polymyxin B-induced rhabdomyolysis was considered.

**Intervention:**

We ceased polymyxin B treatment and monitored the patient daily.

**Outcomes:**

Serum creatine phosphokinase levels returned to normal, myoglobin levels decreased, and muscle pain was significantly alleviated after cessation of polymyxin B. We identified this as a case of polymyxin B-induced rhabdomyolysis.

**Lessons:**

Here, we report the first reported case of rhabdomyolysis induced by polymyxin B administration. The awareness of rare adverse reaction helps ensure the clinical safety of polymyxin B treatment.

## 1 Introduction

Polymyxin B is an effective antibiotic against extensively drug-resistant (XDR), gram-negative bacteria. Polymyxin B binds to the lipopolysaccharide on the membrane of gram-negative bacteria, replacing Mg^2+^ and Ca^2+^, thus destroying the bacterial cell membrane's integrity and increasing permeability.^[1–3]^ Several studies have shown that polymyxin B can strengthen the antibacterial activity of other antibiotics. For example, the combined administration of polymyxin B and meropenem to treat carbapenem-resistant bacterial infections enhances the penetrative ability of meropenem and restores sensitivity.^[4–7]^

At present, nephrotoxicity is the most common side effect of polymyxin B.^[8]^ Besides, some slight adverse reactions, such as pruritus, rash, irritant cough, and skin hyperpigmentation, have been observed.^[9]^ Neurotoxicity caused by polymyxin B is relatively rare. However, abnormal sensory processing and respiratory depression reported in a few cases are known as severe adverse reactions.

Although 2 cases of rhabdomyolysis caused by polymyxin E have been reported,^[10,11]^ such adverse side effects have not been previously reported for polymyxin B. Here, we present a case of rhabdomyolysis in a patient who suffered from pneumonia and bacteremia caused by extensively drug resistance-acinetobacter baumannii and was treated with polymyxin B. This study was approved by the Ethics Committee of Shanghai General Hospital, Shanghai Jiao Tong University, School of Medicine. All data generated or analyzed during this study are included in this published article.

## 2 Case presentation

A 70-year old female, with a 50-year history of aggravating dyspnoea and edema, was admitted to the department of cardiovascular surgery on February 2, 2018. The admission diagnosis was severe mitral valve insufficiency, moderate aortic insufficiency, severe tricuspid regurgitation, and cardiac function IV. She was alert and oriented with the appearance of dyscrasia. She had penicillin allergy but had no alcohol and drug abuse history.

**Figure 1:**
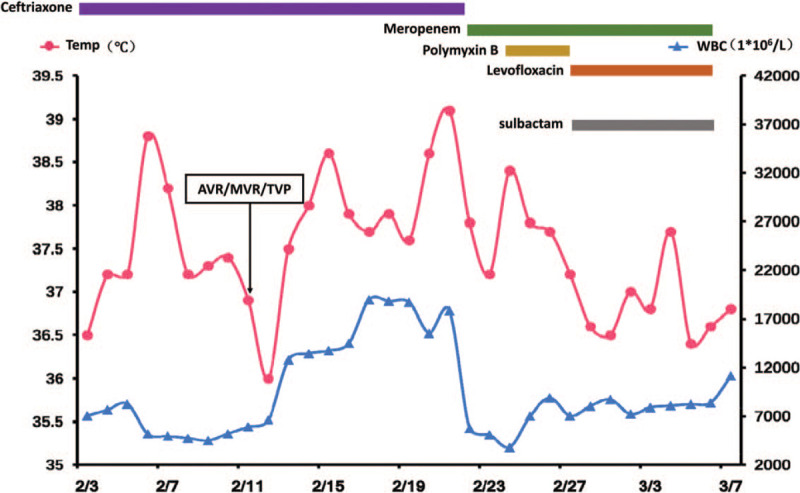
Temperature and white blood cell relative to the duration of medication administration. Temp (red) = body temperature, WBC (blue) = white blood cell.

**Figure 2:**
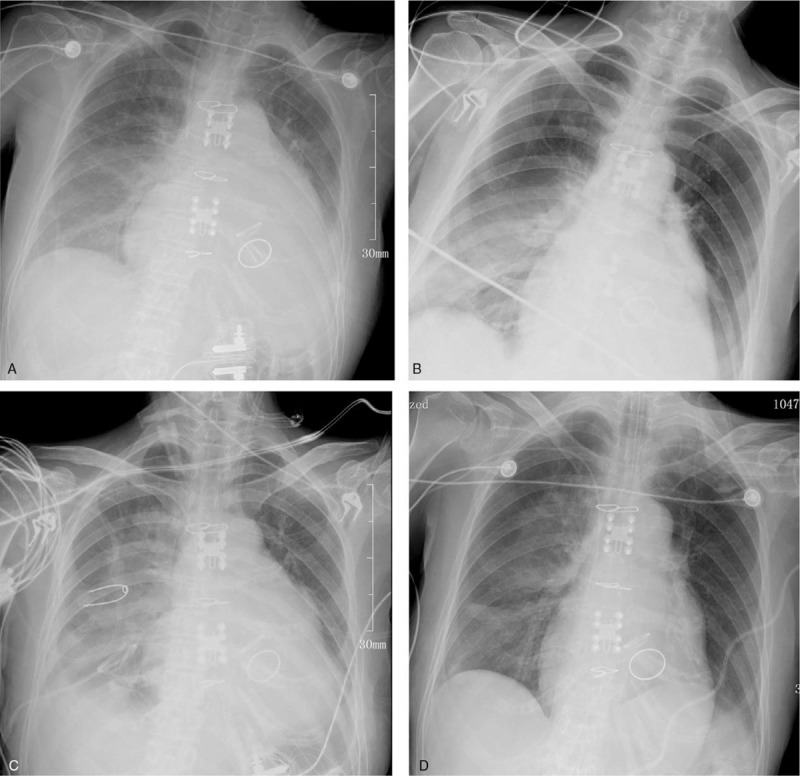
Dynamic changes in chest X-ray during hospitalization. (A). On February 16, the chest X-ray showed that the lung was unclear. (B). On February 22, the chest X-ray presented the consolidation in the right lower lung. (C, D). Following polymyxin B intravenously administered, the lung consolidation dissipated and clear.

**Figure 3:**
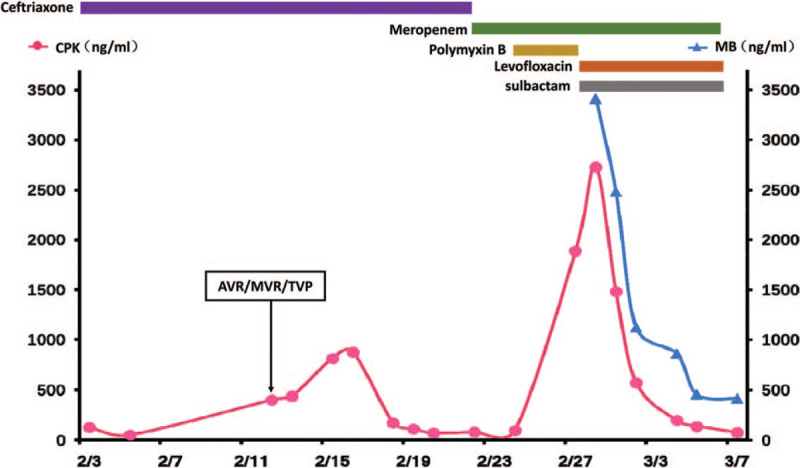
Changes in serum creatine phosphokinase and myoglobin relative to the duration of medication administration. CPK (red) = serum creatine phosphokinase; MB (blue) = myoglobin.

A chest computed tomography scan showed heart enlargement and bilateral pleural effusion. After treatment with inotropic and diuretic drugs, the heart failure symptoms were gradually ameliorated. Mitral valve replacement, aortic valve replacement, and tricuspid valvuloplasty were successfully performed on February 12. Following the operation, the patient's condition was stable, and she was extubated on the third day. Cefuroxime axetil was used as a prophylactic antibiotic (Fig. 1). However, the chest X-ray showed that the lung was unclear (Fig. 2A). Subsequently, the patient exhibited dyspnoea, with abundant white sputum and wet rales in both lungs. As we believed she was experiencing heart failure, we administered non-invasive ventilator assistance and other inotropic treatments. On February 21, the patient was febrile (39.1 °C), accompanied by an elevated white blood cell ( 17.84 × 10^9^/L) count, yellow sputum, and double lung phlegm sounds (Fig. 1). Meropenem (1000 mg Q12 hour) was then administered for empirical treatment. The chest X-ray presented in Figure 2B shows the consolidation in the right lower lung. On February 24, the patient developed fever once again and was intubated. Mechanical ventilation and inotropic support were provided for heart failure. Sputum culture and blood culture revealed a severe acinetobacter baumannii infection. The drug sensitivity test results showed that the bacterium was resistant to carbapenem, ampicillin/sulbactam, and cefepime; moderately sensitive to levofloxacin and minocycline; and sensitive to polymyxin B. Following consultation with an antibiotics expert, polymyxin B (25 mg Q12 hour) was intravenously administered from February 24 combined with meropenem (500 mg Q12 hour). During the following 5 days of antibiotic therapy, the patient's temperature gradually reduced to the normal level, and the pulmonary rales decreased. However, the patient complained of muscle pain and swelling in her limbs, which gradually worsened. serum creatine phosphokinase (CPK) (2730 ng/mL) was significantly increased, compared with February 21 (CPK 68 ng/mL) and February 24 (CPK 94 ng/mL). There was also an emergent high level of myoglobin (MB, 3413.6 ng/mL) at the same time (Fig. 3). Rhabdomyolysis induced by medication was diagnosed, and polymyxin B was considered the cause. Following discontinuation of polymyxin B treatment on February 28, CPK and MB values decreased the next day dramatically. Levofloxacin (600 mg QD) and sulbactam (500 mg Q12 hour) were prescribed, combined with meropenem (500 mg Q12 hour). On March 7, myalgia and myasthenia symptoms were markedly attenuated with a CPK of 73 ng/mL and MB of 419 ng/mL. Fortunately, the renal function remained normal during rhabdomyolysis (creatinine below 100 μmol/L). The lung consolidation dissipated following drainage of the pleural effusion and was subsequently obviously absorbed (Figs. 2C, D). Simultaneously, a red rash appeared on the back and limbs, which gradually subsided following treatment with loratadine. A tracheotomy was performed on March 16, and the patient gradually recovered. The patient was weaned off the ventilator after 56 days and was discharged in June 2018.

## 3 Discussion

To the best of our knowledge, this is the first report of polymyxin B-induced rhabdomyolysis. Rhabdomyolysis is a severe clinical syndrome; its clinical manifestations include muscle swelling, pain, and limited normal activity. Patients with severe systemic symptoms present nausea, vomiting, mental symptoms, and renal failure. Rhabdomyolysis is the result of myocyte injury. Many factors, including trauma, excessive muscular exercise, ischemia, medications, toxins, and hereditary disorders, contribute to mitochondrial damage and plasma membrane rupture in myocytes, leading to an increase in intracellular free ionized calcium, which results in cell death.^[12,13]^ The clinical diagnostic criteria for rhabdomyolysis are: increased CPK (10-fold the normal value) and markedly increased MB levels.^[14–16]^ In this case, the patient exhibited significant weakness, mild muscle soreness, and markedly increased CPK and MB levels after using polymyxin B for 5 days. Once polymyxin B was withdrawn, the myalgia and myasthenia symptoms were alleviated, and the CPK and MB values rapidly decreased. These symptoms are simultaneous with polymyxin B administration. The patient was subsequently treated with meropenem combined with levofloxacin and sulbactam; her CPK and MB levels returned to normal, and the myasthenia was alleviated. In terms of rhabdomyolysis, inducements like muscle injury, limb ischemia, statin, daptomycin, or interferon were not present in this case. There have been reports of meropenem or levofloxacin related rhabdomyolysis.^[17,18]^ But meropenem was prescribed before and after the occurrence of rhabdomyolysis and showed no effect on rhabdomyolysis. Levofloxacin was applied after the withdrawal of polymyxin B and continued while CPK descended to normal level. Oppositely, there was a chronological presence of polymyxin B and myopathy symptoms, and the patient quickly recovered from rhabdomyolysis after discontinuing of polymyxin B. Therefore, the adverse reaction observed in this case was thought to be rhabdomyolysis induced by polymyxin B.

In this case, we hypothesized that the mechanism underlying rhabdomyolysis might be related to mitochondrial injury or neurotoxicity, based on several reports.^[19–21]^ In rat models, polymyxin B assembles around the mitochondria and endoplasmic reticulum of renal tubular cells, leading to the activation of caspase –3, –8, and –9.^[19]^ Another study about polymyxin B related nephrotoxicity demonstrated that polymyxin B damaged mitochondria by inducing their fragmentation, reducing membrane potential, and stimulating oxidative activities in kidney proximal tubular cells.^[20]^ Ahmed et al found that polymyxin B injured mitochondria in lung epithelial cells.^[21]^ In terms of neurotoxicity, polymyxin B could potentially act on the presynaptic membrane of nerve cells and inhibit acetylcholine release, leading to dysfunction of nerve-muscle junction transmission. Long-term depolarization of neural cells caused by polymyxin B could induce calcium overload in muscle cells and cause cell death.^[9,22]^ Besides, polymyxin B neurotoxicity is generally reversible, and muscle would recover in a short time.^[23]^

Currently, polymyxin B is used to treat gram-negative bacteria and exhibits superior antibacterial activity in clinical settings. The current consensus is polymyxin B should be prescribed by body weight (2.0–2.5 mg/kg as a loading dose on, followed by 1.25–1.5 mg/kg every 12 hours) and adjusted according to renal function.^[24]^ As nephrotoxicity is associated with polymyxin B exposure, some researchers suggested checking blood concentration to optimize dosing, especially in critically ill patients.^[25]^ In this case, the dosage of polymyxin B was less than 1 mg/kg every 12 hours with normal renal function. Regrettably, an assay of polymyxin B plasma concentration was not available at that time. We suppose rhabdomyolysis was not related to an overdose. However, existing research data remain limited; the medication regimen and toxicological characteristics require more comprehensive and thorough investigation. This first case of rhabdomyolysis also indicates that doctors and clinical pharmacists should be very concerned about the use of polymyxin B. Renal toxicity, as well as other discomforts during the treatment period, should be monitored carefully and reported promptly to ensure the clinical safety of polymyxin B and optimize the treatment regimen.

## Acknowledgments

The authors would like to thank the patient's family for giving consent.

## Author contributions

**Conceptualization:** Min Yu, Sheng Shi.

**Data curation:** Ming Ni, Yanan Zhao, Limin Wang.

**Funding acquisition:** Min Yu.

**Investigation:** Ming Ni, Xiangdong Meng, Limin Wang.

**Project administration:** Limin Wang, Min Yu, Sheng Shi.

**Supervision:** Min Yu, Sheng Shi.

**Validation:** Min Yu, Sheng Shi.

**Writing – original draft:** Ming Ni.

**Writing – review & editing:** Ming Ni, Yanan Zhao.
